# Interfering Nuclear Protein Laminb1 Induces DNA Damage and Reduces Vemurafenib Resistance in Melanoma Cells In Vitro

**DOI:** 10.3390/cancers16234060

**Published:** 2024-12-04

**Authors:** Yuan Li, Yuqing Feng, Dan Chen

**Affiliations:** Bioinspired Engineering and Biomechanics Center (BEBC), Xi’an Jiaotong University, Xi’an 710049, China; fyq20230628@stu.xjtu.edu.cn (Y.F.); chendan@stu.xjtu.edu.cn (D.C.)

**Keywords:** laminb1, melanoma, BRAF inhibitors, DNA damage

## Abstract

Background/Objectives: Drug resistance poses a substantial clinical challenge in melanoma treatment, yet the underlying mechanism remains elusive. Here, we report the novel role of laminB1, a nuclear structure protein, in regulating the response of BRAF-mutated melanoma cells to vemurafenib. Results: Our analysis of clinical samples and existing databases highlights the tight correlation between the laminB1 expression level and melanoma progression and prognosis. Notably, we observe that laminB1 expression is upregulated when BRAF-mutated melanoma cells develop resistance to vemurafenib. The knockdown of laminB1 substantially increases the sensitivity of melanoma cells to vemurafenib. Furthermore, we found laminB1 suppression increases cell apoptosis via the escalation of DNA damage in a vemurafenib-dose-dependent manner. Conversely, protective cell autophagy is negatively regulated by laminB1 suppression. Interestingly, this distinct regulation pattern of apoptosis and autophagy by laminB1 cooperatively promotes the response of BRAF-mutated melanoma cells to vemurafenib. Conclusions: Our findings unveil the potential of laminB1 as both a diagnosis marker and a therapeutic target of melanoma.

## Simple Summary

Drug resistance remains a significant challenge in melanoma treatment. This study identifies laminB1, a nuclear structural protein, as a key regulator of melanoma cell response to target therapy. LaminB1 expression tightly relates to melanoma progression and prognosis. Interfering laminB1 amplifies the drug response of melanoma cells by a cooperative effect of enhanced apoptosis and suppressed protective autophagy. These findings highlight the potential of laminB1 as a diagnostic marker and therapeutic target for melanoma treatment.

## 1. Introduction

Melanoma, one of the most aggressive forms of skin cancer, is associated with BRAF gene mutations in roughly 50% of cases, leading to the persistent activation of the BRAF gene. The initial targeted therapy, treatment with a BRAF inhibitor (BRAFi), yields a remarkable response rate (exceeding 80%) in patients. However, after treatment within a certain time (around 6–12 months), most patients lose their responsiveness and ultimately develop resistance to targeted therapy. Several mechanisms have been identified to explain the occurrence of BRAFi resistance [1], including the activation of alternative survival pathways [2], increased androgen receptor expression [3], and elevated secretion of collagenase [4].

Recently, emerging evidence underscores the importance of crosstalk between apoptosis and autophagy in cancer [5]. In the early stages of oncogenesis, autophagy prevents cancer progression by degrading and eliminating misfolded proteins and damaged organelles [6], whereas apoptosis is one of the major pathways of cancer cell death [7]. Interestingly, along with cancer progression or drug treatment, autophagy not only synergizes with drug-induced tumor cell death but also resists drug-induced apoptosis [8]. Several regulators have been reported to regulate autophagy and apoptosis cooperatively. For example, the B-cell lymphoma-2 family protein has been demonstrated to initiate and regulate autophagy [9,10] and apoptosis [11,12] separately, through interactions with distinct ligands. This crosstalk between autophagy and apoptosis has been implied to determine the response of cancer cells to targeted therapy. For example, AKT/mTOR signaling is involved in both apatinib-induced autophagy and apoptosis in anaplastic thyroid carcinoma, and inhibiting autophagy increases drug-induced apoptosis [13]. Similarly, anti-VEGF (bevacizumab) treatments induce both apoptosis and autophagy of colorectal cancer cells, and the inhibition of autophagy promotes apoptosis and, therefore, inhibit cell proliferation [14]. Hence, exploring the underlying regulatory mechanism of apoptosis–autophagy crosstalk is particularly important for anticancer therapy.

As a nucleoskeleton protein, laminB1 is essential to sustain the structural integrity of the nucleus [15]. LaminB1 is shown to physically connect with chromatin via multiple linker proteins, such as lamin B receptors (LBRs) and lamina-associated domains (LADs) [16], and therefore sustains the stability of chromatin and prevents the consequent accumulation of DNA damage resulting from deficient DNA damage repair [17]. Meanwhile, laminB1 can protect RAD51, a classical DNA damage repair factor, from proteasome degradation, enhancing DNA damage repair and therefore reducing apoptosis [18,19]. Recently, the role of laminB1 in the regulation of autophagy has been gradually explored. For example, in cervical cancer cells, laminB1 deletion attenuates the autophagy capacity of cancer cells, thus allowing the easier invasion and proliferation of human papillomavirus [20]. LaminB1 can also act as a substrate for autophagy to induce senescence in osteoarthritic chondrocytes [21]. Interestingly, BRAFi has been demonstrated to induce autophagy and apoptosis in melanoma cells, and its cytotoxicity to cancer cells gradually diminishes once the level of DNA damage repair or autophagy approaches a certain level [22,23]. Although laminB1 can influence apoptosis and autophagy, its role in apoptosis–autophagy crosstalk, especially in the BRAFi administration of melanoma, remains elusive.

Herein, our study uncovers the novel role of laminB1 in regulating the response of BRAF-mutated melanoma cells to BRAFi PLX4720 (vemurafenib). The analysis of clinical samples and databases reveals a strong link between laminB1 expression and melanoma progression. LaminB1 is upregulated in PLX4720-resistant cells, and its knockdown significantly enhances drug responsiveness. LaminB1 suppression increases apoptosis in a dose-dependent manner, while negatively regulating protective autophagy. The coordinated regulation of apoptosis and autophagy by laminB1 improves the response to PLX4720, positioning laminB1 as a potential therapeutic target in overcoming melanoma drug resistance.

## 2. Materials and Methods

### 2.1. Cell Culture

The human melanoma cells WM115 (iCell Bioscience, Shanghai, China) were cultured in MEM Alpha Modification (Hyclone, Logan, UT, USA), supplemented with 10% FBS (Gibco, Waltham, MA, USA) and 1% Penicillin/Streptomycin (Hylcone, Logan, UT, USA). WM164 cells (a gift from Dr. Songmei Geng’s lab) were cultured in RPMI 1640 (Corning, Corning, NY, USA) supplemented with 10% FBS (Gibco, Waltham, MA, USA) and 1% Penicillin/Streptomycin (Hylcone, Logan, UT, USA). All cells were maintained at 37 °C in a humidified 5% CO_2_ environment with passage at 80% confluence. All melanoma cells acquired resistance to PLX4720 after 1–2 months of chronic treatment (5μM) until no cell death was observed. Resistant cells were designated WM115R and WM164R.

### 2.2. Drug Treatments

PLX4720 (Vemurafenib) was purchased from Cell Signaling Technology (CST, Danvers, MA, USA) and dissolved in DMSO to 10 mM as a stock solution. The PLX4720 stock solution was added to culture media to prepare the work media with the required concentration (1 μM, 15 μM, 50 μM, etc.), and media with the same concentration of DMSO were prepared as the control group. The treatment time interval was 3 days for the Vemurafenib treatment. The cell survival after treatment was assessed by an MTT (MCE, Monmouth Junction, NJ, USA) assay.

### 2.3. Lentivirus Transduction

Short hairpin RNAs (shRNA) lentivirus against laminB1 (TRCN-0000029271) were purchased from Genechem (Shanghai, China). Viral transduction was conducted onto primary melanoma cells following the manufacturer’s protocol. Protein samples were collected 48 h after transfection and a western blot assay was used to verify the knockdown efficiency.

### 2.4. Real-Time PCR

According to the instructions of the kit, the Trizol kit was used to extract the total RNA from melanoma cells in each group. Micro ultraviolet spectrophotometry was used to detect the RNA concentration and purity. According to the instructions in the SYBR PremixExTaq II kit, the reaction conditions were set to 95 °C for 10 s, 95 °C for 5 s, and 60 °C for 30 s (40 cycles). The relative expression level of the target gene is expressed as 2^−△△Ct^. The sequence of primers used is shown as the following:

GAPDH (F: GTCTCCTCTGACTTCAACAGCG, R: ACCACCCTGTTGCTGTAGCCAA);

LB1_1 (F: AAGCAGCTGGAGTGGTTGTT, R: TTGGATGCTCTTGGGGTTC);

LB1_2 (F: CTGCTGCTCAATTATGCCAAGAAG, R: GGCAGATAAGGATGCTTCTAGCT).

### 2.5. Western Blotting

The extracted protein samples were boiled and denatured in an RIPA lysis buffer (Beyotime, Shanghai, China) after extraction, and the amount of protein was determined by a BCA assay kit (Epizyme, Shanghai, China). Protein with equal mass was loaded into polyacrylamide gels and separated by electrophoresis. The polyvinylidene difluoride membranes were blocked in a blocking buffer (Epizyme, Shanghai, China) at room temperature for 2 h after the transfer of protein onto membrane. The membranes were then incubated with the primary antibody at 4 °C overnight. After washing the PBST membrane, the membrane was incubated with the secondary antibody at room temperature for 2 h. After washing by TBS with 1% Tween (TBST), the membranes were incubated with HRP-labeled goat anti-mouse/rabbit secondary antibodies for 30 min at room temperature. Once the membrane was developed, images were collected using a chemiluminescence imaging system. The grayscale values of the bands were analyzed and calculated using ImageJ software (https://imagej.net/software/fiji/, 8 January 2022) for each group. Target proteins were probed with the following antibodies: anti-GAPDH (Santa Cruz Biotechnology, Dallas, TX, USA, C65), anti-LaminB1(Cell Signaling Technology, CST, Danvers, MA, USA, 13435S), anti-LC3 (Proteintech, Wuhan, China, 14600-1-AP), anti-Cleaved PARP (Asp214) (Cell Signaling Technology, CST, Danvers, MA, USA, 5625S), anti-Phospho-Histone H2A.X (Ser139) (Cell Signaling Technology, CST, Danvers, MA, USA, 9718S), and anti-LC3 (Proteintech, 81004-1-RR).

### 2.6. Immunohistochemistry

The laminB1 expression in melanoma and melanocytic nevi of the identical melanoma patient tissues was verified by immunohistochemistry. Tissue samples were dewaxed by immersion in a dewaxing solution and 95% ethanol, followed by heating in a microwave oven for antigen recovery. After blocking with 5% goat serum, anti-laminB1 (CST, Danvers, MA, USA) was used for the immunohistochemical analysis. Tissue specimens were incubated with the laminB1 antibody for 10 h at 4 °C and with the secondary antibody for 60 min at room temperature. The immunoreactivity was observed by incubating with diaminobenzidine for 5 min. The cells were visualized with 40 × magnification. The staining effect was observed by microscopy and counted, and the results were interpreted.

### 2.7. Immunofluorescence

In order to assess the DNA repair in the laminB1 knockdown WM115 and WM164 melanoma cells, γ-H2AX foci accumulation, a marker for the appearance of double-strand breaks (DSBs), was detected by the immunostaining technique. The determination of the γ-H2AX protein by WB was conducted at 24 h. Cells were inoculated into 6-well plates with pre-treated coverslips, and cells were allowed to crawl for 24 h. Cells were fixed with 4% paraformaldehyde for 20 min at room temperature and washed with PBS 3 times for 5 min each time, and the membranes were ruptured with PBS containing 0.2% TritonX-100 for 15 min at room temperature and then washed with PBS. Two to three drops of goat blood serum were placed on the crawl piece and incubated at room temperature for 45 min. The anti-γ-H2AX primary antibody, which had been centrifuged and mixed well beforehand, was added, and incubated overnight at 4 °C in a wet box. The samples were then washed three times with PBS containing 0.2% Tween-20 for 5 min each. The fluorescent-labeled secondary antibody was then added and incubated at 37 °C for 45 min while protected from light. The samples were washed again with PBS. DAPI solution was added dropwise, and the staining was performed at room temperature for 5 min, then washed three times with PBS containing 0.2% Tween-20 and finally sealed with 90% glycerol with the cell side facing down. The staining results were observed under a fluorescence microscope.

### 2.8. Bulk RNA Sequencing and Data Analysis

The total RNA was isolated from the WM115 with and without the transfection of the laminB1 lentivirus for 24 h, following the protocol provided by the lentivirus manufacture. Libraries for the RNA sequencing (RNA-seq) were sequenced on an Illumina NovaSeq 6000 platform. RNA-seq data were processed by the OmicStudio tools at https://www.omicstudio.cn/tool, accessed on 11 August 2023.

### 2.9. Statistical Analyses

Statistical analyses were performed by a two-tailed Student’s *t*-test. *p* <  0.05 (*) was considered statistically significant. The results were shown as the mean ± standard error of the mean (SEM). The specific statistical analyses are described in the corresponding figure legend.

## 3. Results

### 3.1. Elevated LaminB1 Expression Has Tight Clinical Relevance with Melanoma Progression

**Figure 1:**
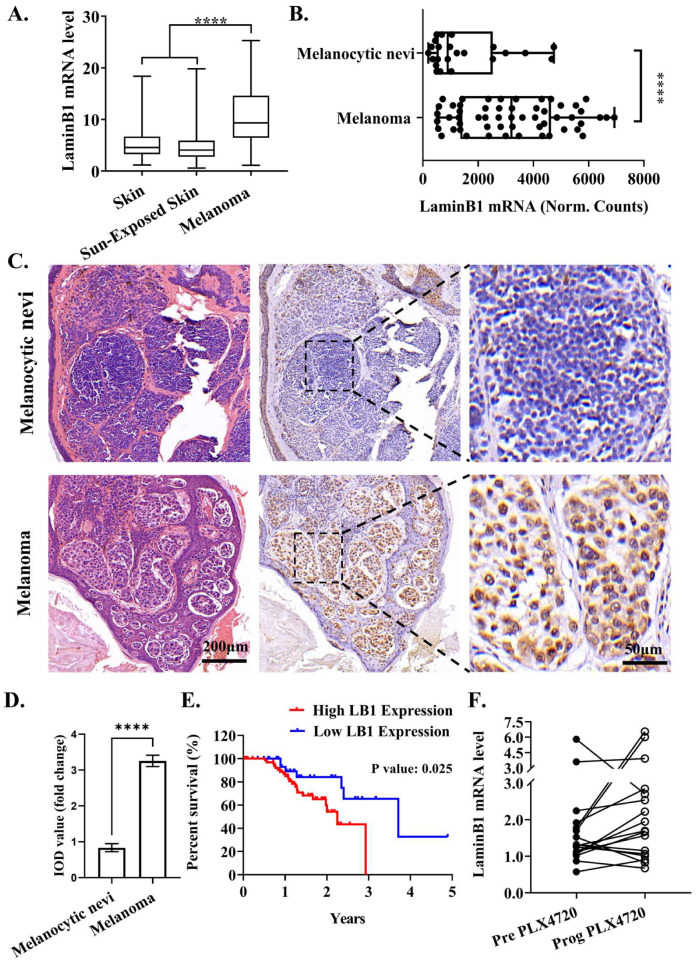
Elevated laminB1 expression has clinical relevance for melanoma. (**A**) The mRNA level of laminB1 in the skin, sun-exposed skin, and melanoma. (**B**) The mRNA level of laminB1in melanocytic nevi and melanoma. (**C**) Immunohistochemistry staining of laminB1 in melanocytic nevi and melanoma, scale bar: 200 μm. (**D**) The IOD value of laminB1 in (**C**). (**E**) Significant differences in survival curves between the high laminB1 expression group (red line) and the low laminB1 expression group (blue line). Data are compared by log-rank test, *p* = 0.025. (**F**) The application of PLX4720 had a significant effect on the laminB1 mRNA level. Data are compared by a two-tailed Student’s *t*-test and **** indicates *p* < 0.0001.

To identify the clinical correlation between laminB1 and melanoma, we first searched the Gene Expression Omnibus (GEO) database. Compared to normal skin tissue with and without sun exposure, the melanoma tissue had a significantly higher level of laminB1 mRNA (Figure 1A). Specific to melanocytic nevi, the mRNA level of laminB1 also increased dramatically with the occurrence of melanoma (Figure 1B). Therefore, the upregulation of laminB1 is closely related with melanoma development. Next, we conducted histological assessments on melanoma patients to validate this correlation further. Consistently, the expression level of laminB1 was upregulated in melanoma compared to melanocytic nevi of the identical melanoma patient, shown through histological analysis (Figure 1C,D). Eventually, we collected and analyzed the prognosis data of patients from the TCGA database to assess the upregulation of laminB1 on patient prognosis (https://www.proteinatlas.org/ENSG00000113368-LMNB1/cancer/melanoma, accessed on 20 October 2023). The median survival time (MST) of high-laminB1-expression (HLB) patients was 1.98 years (95%CI: 0.17 to 1.63); in contrast, a low-laminB1-expression (LLB) patient had an MST of 3.71 years (95%CI: 0.61 to 5.74) (Figure 1E). Meanwhile, 40% of LLB patients had an overall survival (OS) of over 5 years; however, no HLB patients had an OS of over 3 years (Figure 1E). Moreover, we reanalyzed the RNA sequence data of the GEO database, and found that 65% of the BRAF-mutation melanoma patients (11 out of 17) gave rise to an upregulated mRNA level of laminB1 after treatment of BRAFi, PLX4720 (Figure 1F). In sum, the expression level of laminB1 tightly relates to melanoma progression, prognosis, and response to targeted therapy.

### 3.2. LaminB1 Positively Correlates with the Response of Melanoma Cells to PLX4720

**Figure 2:**
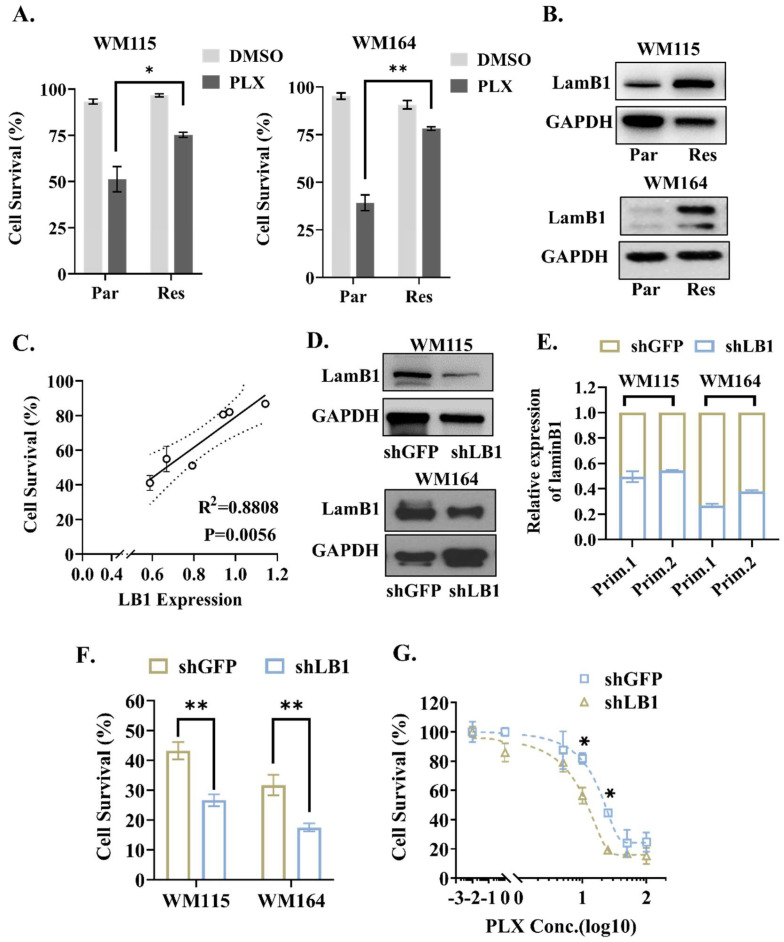
LaminB1 positively correlates with the response of melanoma cells to targeted therapy. (**A**) Quantitative analysis and comparison of survival rates of WM164 and WM115 cells treated with 15μM PLX4720 and DMSO, respectively. “Par” and “Res” indicate the parental cell and resistant cell, respectively. Data are compared by a two-tailed Student’s *t*-test and * indicates *p* <  0.05, ** indicates *p* <  0.01. (**B**) Western blot analysis of the expression and quantification of laminB1 in WM115 and WM164 cells after PLX4720 treatment; GAPDH was used as a control. “Par” and “Res” indicate the parental cell and resistant cell, respectively. (**C**) A positive linear correlation exists between LB1 expression level and cell survival rate. (**D**) Western blot analysis of the expression and quantification of laminB1 in shGFP and shLB1 of WM115 and WM164; GAPDH was used as a control. (**E**) Relative expression and quantitative analysis of laminB1 in shLB1 and shGFP of WM115 and WM164 cells. (**F**) Quantitative analysis of cell survival of shLB1 and shGFP of WM115 and WM164. Data are compared by a two-tailed Student’s *t*-test and ** indicates *p* <  0.01. (**G**) Quantitative analysis of cell survival and PLX4720 concentration (μM) in shLB1 and shGFP. Data are compared by a two-way ANOVA and Holm-Sidak test and * indicates *p* <  0.05. Original western blots are presented in File S1.

We established two BRAF-mutated melanoma cell lines (WM115 and WM164) with PLX4720-resistance to further validate the correlation between the laminB1 expression and the response to PLX4720. After the chronic treatment of PLX4720, the successful construction of chemoresistance was confirmed by a 50% to 100% increased survival of resistant cells compared to parental cells, which is due to the lack of PLX4720 resistance (Figure 2A). The expression level of laminB1 appears elevated after the melanoma cell acquired chemoresistance (Figure 2B). Meanwhile, the expression level of laminB1 had a strong correlation (R2 = 0.88) with the post-PLX4720 survival of the melanoma cell, independent of the types of cell line and chemoresistance level of the cell (Figure 2C). Then, We suppressed the expression of laminB1 in the parental melanoma cell lines via the transfection of lentivirus shRNA, to assess the influence of laminB1 on the PLX4720 resistance of the melanoma cell (Figure 2D,E). Both cell lines showed approximately a 50% reduction in cell survival with 15 μM PLX4720 treatment after laminB1 reduction (Figure 2F). In addition, we conducted a dose-dependent response assay to assess the dependence of the melanoma PLX4720 response on laminB1. For most doses of the PLX4720 treatment, the laminB1 knockdown group responded better than the control group, but a significant difference appeared among the range from 10 μM to 25 μM (Figure 2G). The IC50 value of PLX4720 dropped from 20 μM to 12 μM after the interference of the laminB1 expression with shRNA (Figure 2G). Therefore, the expression level of laminB1 determines the responsiveness of melanoma cells to targeted therapy.

### 3.3. Loss of LaminB1 Promotes PLX4720-Induced Apoptosis in Melanoma Cells via Accumulation of DNA Damage

**Figure 3:**
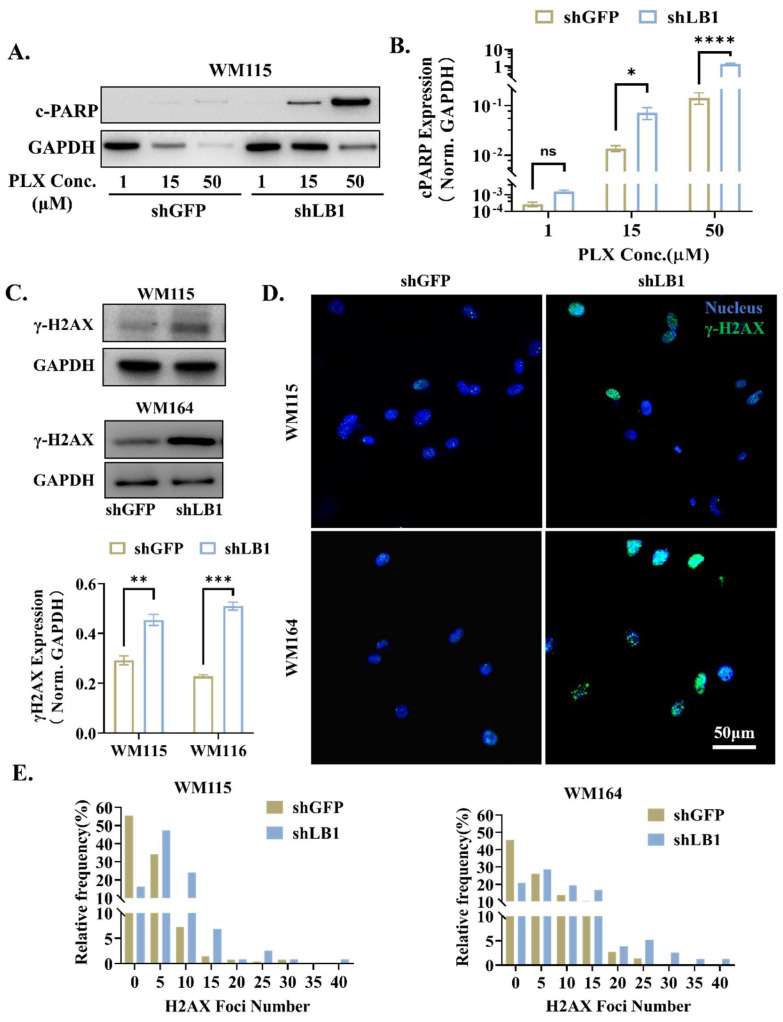
LaminB1 knockdown induces cell apoptosis via DNA damage. (**A**) Western blot analysis of c-PARP expression and quantification at different PLX concentrations; GAPDH was used as a control. (**B**) Quantitative analysis of c-PARP expression in shLB1 and shGFP at different PLX4720 concentrations; GAPDH was used as a control. Data are compared by two-way ANOVA and Holm-Sidak test and * indicates *p* <  0.05, **** indicates *p* <  0.0001. (**C**) Western blot analysis of expression and quantification of r-H2AX in shGFP and shLB1 of WM115 and WM164, respectively; GAPDH was used as a control. Data are compared by a two-tailed Student’s *t*-test and ** indicates *p* <  0.01, *** indicates *p* <  0.001. (**D**) Immunofluorescence of γ-H2AX inWM115 and WM164 cells of shGFP and shLB1 groups (green, γ-H2AX; blue, DAPI). Scale bar: 50 μm. (**E**) Quantitative analysis of the distribution of H2AX foci number in shGFP and shLB1 of WM115 and WM164. Original western blots are presented in File S1.

Next, we examined the apoptotic level of melanoma cell lines with laminB1 interference, since apoptosis is the essential reason for cell death when treated with targeted therapy. The expression level of the apoptotic marker c-PARP increased dramatically, along with the augmenting treatment (Figure 3A,B). Compared to the control group, the knockdown of laminB1 significantly increased the expression level of c-PARP (Figure 3A,B). We next examined the level of DNA damage of melanoma cell lines with laminB1 interference, since targeted therapy typically induces DNA damage and, therefore, kills cancer cells. For both WM115 and WM164 cell lines, the laminB1 knockdown significantly increased the appearance of γ-H2AX, a classical marker of DNA damage (Figure 3C). The quantification of the γ-H2AX number per cell gave a clear shift of distribution towards a higher value (Figure 3D). Meanwhile, the percentage of cells with the number of γ-H2AX foci over 10 (an indicator of cell DNA damage) in the nucleus increased from 10.5% to 36.2% (WM115) and 28.3% to 50.6% (WM164), indicating the laminB1 knockdown escalated the occurrence of DNA damage. This is further confirmed by a roughly two-fold increase in γ-H2AX expression in cells with laminB1 knockdown (Figure 3E). Hence, laminB1 protects melanoma cells from targeted therapy-induced cell death via suppressing apoptosis.

### 3.4. Regulatory Role of LaminB1 in Autophagy Is Unveiled by Bioinformatics Analysis in Melanoma Cells

**Figure 4:**
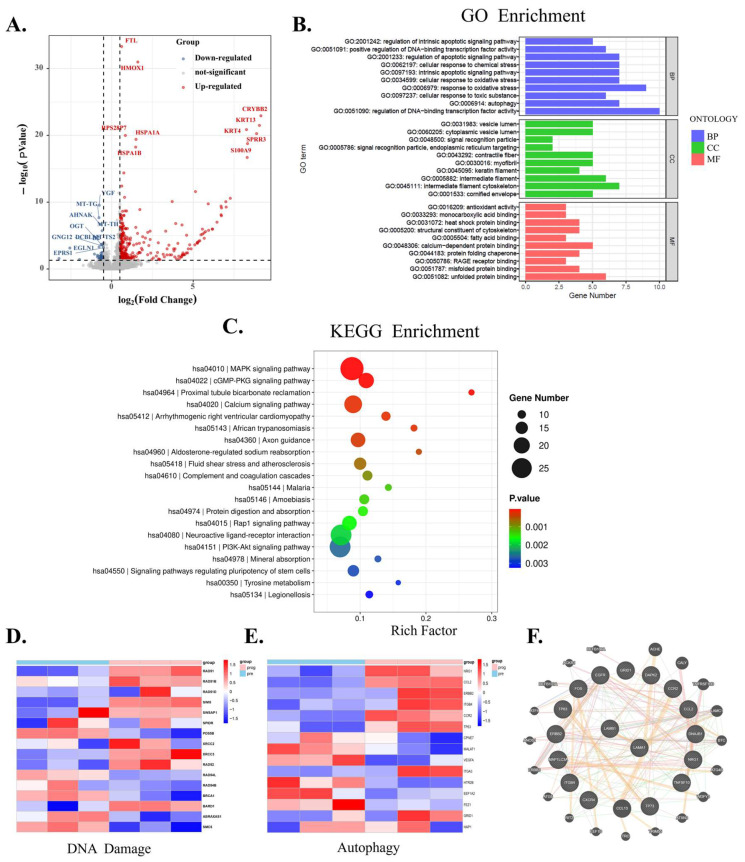
Bioinformatics analysis of autophagy regulation by laminB1. (**A**) The Volcano plot of differentially expressed genes (DGEs). (**B**) GO Terms Enrichment analysis of DGEs. (**C**) KEGG pathway enrichment analysis of DGEs. (**D**) Heat map of differentially expressed genes of DNA-damage-related genes. (**E**) Heat map of differentially expressed genes of autophagy-related genes. (**F**) Interaction map of laminB1 with other proteins.

LaminB1 has been reported to influence cell autophagy, so we conducted an RNA seq analysis of melanoma cells WM115 without and with laminB1 knockdown by shRNA, to assess its impact on autophagy regulation. Our screening results showed that there was a total of 360 significantly differentially expressed genes (DEGs) in the laminB1 knockdown group compared to the control group (Figure 4A). The number of upregulated genes (red) and downregulated genes (blue) were 310 and 30, respectively, as shown in the volcano plot. To explore the biological functions and pathways involved in DEGs, we subsequently analyzed DEGs for both GO pathway enrichment (Figure 4B) and KEGG pathway enrichment (Figure 4C), respectively. In the molecular function (MF) section of the GO plot (Figure 4B), we observed a significant enrichment of GO terms associated with the regulation of intrinsic apoptotic signaling pathway, cellular response to chemicals, oxidative stress, and autophagy. In the cellular component (CC) section of the GO plot (Figure 4B), several GO terms related to autophagy and apoptosis, such as cytoskeleton and cellular contractility, appear significantly enriched. Regarding the biological process (BP), antioxidant activity, calcium-dependent protein binding, and RAGE receptor binding, which closely relate to autophagy and apoptosis, are shown to be significantly enriched. Moreover, the KEGG analysis showed that DEGs were significantly enriched in MAPK, PI3K-AKT, and Rap1 signaling pathways, which are associated with cell autophagy (Figure 4C). We then further analyzed several presentative genes of DNA damage repair (Figure 4D) and autophagy (Figure 4E) and found their expression pattern changed after laminB1 knockdown. Finally, we graphed the protein interactions of laminB1 and found it interacts with many proteins that regulate autophagy and DNA damage (Figure 4F). Therefore, laminB1 transcriptionally participates in the regulation of autophagy and apoptosis.

### 3.5. LaminB1 Is Essential to the Crosstalk Between Apoptosis and Autophagy in Responding to PLX4720

**Figure 5:**
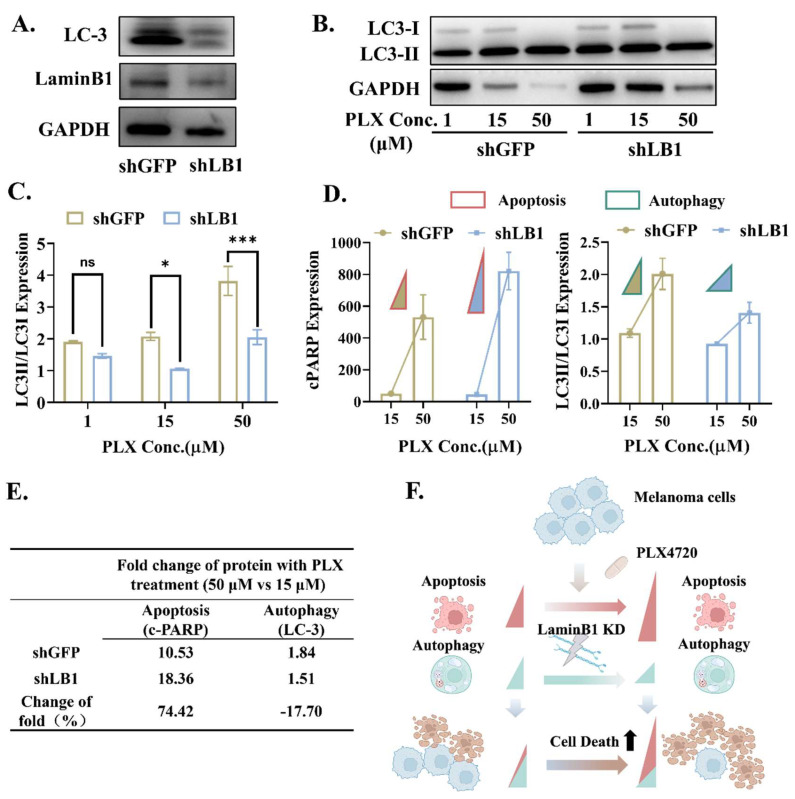
The different regulation patterns of laminB1 on autophagy and apoptosis. (**A**) Western blot of LC-3 and LaminB1expression in shGFP and shLB1; GAPDH was used as a control. (**B**) Western blot analysis for quantitative analysis of LC3-I and LC3-II expression at different PLX 4720 concentrations in shLB1 and shGFP; GAPDH was used as a control. (**C**) Quantitative analysis and comparison of LC3-I/LC3-II expression ratio at different PLX4720 concentrations in shGFP and shLB1. Data are compared by two-way ANOVA and Holm-Sidak test and * indicates *p* <  0.05, *** indicates *p* <  0.001. (**D**) Quantitative analysis and comparison of c-PARP expression and LC3-I/LC3-II expression ratio at different PLX4720 concentrations in shGFP and shLB1. (**E**) Table: fold change in protein with PLX4720 treatment (50 vs. 10 μM). (**F**) Illustrative images of our proposed mechanism. Original western blots are presented in File S1.

Consistent with the bioinformatics analysis, the expression of autophagy-related proteins LC3 was downregulated by laminB1 knockdown (Figure 5A). Meanwhile, the post-PLX4720 change in the representative indicator of autophagy, with a ratio of LC3-II/LC-3I, showed a dose-dependent manner for both control and laminB1 knockdown groups (Figure 5B,C). In contrast to the control, laminB1 knockdown reduced the PLX4720-induced autophagy, and this suppressive effect intensifies with the increased dose of PLX4720, with a 23% reduction for 1 μM, 48% reduction for 15 μM, and 46% reduction for 50 μM (Figure 5B,C). This indicates that laminB1 is possibly essential to maintain the proper responsive machinery of autophagy in the presence of a pro-autophagic stimulus. The suppressive role of laminB1 knockdown was completely distinct from its promotive role on apoptosis (Figure 3A,B). The similarity is that the effect of laminB1 loss on both autophagy and apoptosis was enhanced along with increases in PLX4720 dose, as shown in the normalized change with the corresponding value in groups with the 1μM PLX4720 treatment (Figure 5D). Regardless of the laminB1 interference, the PLX4720 treatment increased the level of apoptosis more than the level of autophagy (10.53 folds vs. 1.84 folds for control, and 18.36 folds vs. 1.51 folds for laminB1 knockdown group) (Figure 5E). Although the knockdown of laminB1 still increased the PLX-4720-induced apoptosis (18.36 folds) and autophagy (1.51 folds), the change trend was completely different. The positive change (74.42%) in the folds in apoptosis indicates the suppressive role of laminB1 on apoptosis, while the negative change in folds (−17.7%) in autophagy indicates the protective role of laminB1 on autophagy (Figure 5E). Hence, the knockdown of laminB1 enhanced the response of the BRAF-mutated melanoma to PLX4720 through the cooperative regulation of apoptosis enhancement and autophagy suppression (Figure 5F).

## 4. Discussion

Autophagy and apoptosis play crucial roles in oncogenesis, cancer progression, and cancer therapy. They can synergistically induce cancer cell death, while protective autophagy in cancer cells can also inhibit apoptosis induced by anticancer drugs [24]. Thus, recent studies have emphasized the importance of the crosstalk between autophagy and apoptosis in cancer drug response. For example, autophagy protein 1 (Ambra1) is located at the translational crossroads between autophagy and apoptosis; its expression and distribution regulate the homeostasis between autophagy and apoptosis, thereby altering the efficacy of cancer treatment [25,26]. The anti-apoptotic BCL2 family proteins have also been shown to regulate the crosstalk between autophagy and apoptosis, determining the response of cancer cells to therapy [27,28]. Most studies focus on the molecular signaling pathways or transcription factors in regulating crosstalk. In the present study, we uncover the novel role of the nucleoskeleton protein laminB1 in mediating this crosstalk, although laminB1 is commonly recognized to cooperate with other types of lamin to provide the nucleus with mechanical support. More interestingly, the regulation of laminB1 on apoptosis and autophagy appears opposite, but these distinct regulations can cooperate to determine the drug responsiveness of cancer cells.

Many studies have demonstrated that protective autophagy induced by cancer drugs can free cancer cells from drug-induced apoptosis. For example, cisplatin induces protective autophagy in bladder cancer cells, and the combination of autophagy inhibitors enhances the sensitivity of cancer cells to cisplatin [29]. Similarly, our study shows that deficient laminB1 substantially reduces the increased protective autophagy against escalated apoptosis after BRAFi treatment. This is further supported by our RNA-seq data, showing that negatively regulated genes of autophagy, such as NRG1, CCR2, and ERBB2, are upregulated by laminB1 knockdown, and vice versa. Although we have demonstrated the role of laminB1 in maintaining the upregulation of post-BRAFi autophagy, the detailed mechanism still needs further exploration.

The BRAF gene is associated with cell proliferation and growth, and approximately 40% to 60% of melanoma patients have BRAF V600 mutations. BRAFi is a targeted therapy for melanoma patients with mutations in the BRAF gene, and its use in the clinic has made good progress, but resistance due to long-term dosing remains a clinical challenge [30]. Therefore, it is often combined with other therapies, such as MEK inhibitors (e.g., trametinib), to improve efficacy and delay the development of resistance [31]. The potential of combined autophagy modulators (e.g., chloroquine, hydroxychloroquine, rapamycin, etc.) in the treatment of malignant cancers has also attracted attention, as a growing number of studies have identified the role of aberrant autophagy in BRAFi resistance [32]. The upregulation of autophagy induced after treatment with BRAFi has been confirmed in melanoma, accompanied by a significant decrease in response rate and shortened progression-free survival with BRAFi, and in vitro experiments have demonstrated that the use of autophagy inhibitors overcomes the resistance of melanoma cell lines [22,33]. Recent studies have shown that BRAFi, in combination with the autophagy inhibitor chloroquine, reverses clinical presentation and imaging progression in glioma patients [34,35]. Therefore, the application of targeted therapy combined with autophagy modulators in the clinic is just around the corner.

## 5. Conclusions

In conclusion, we unveil that the distinct regulatory patterns of laminB1 on apoptosis and autophagy cooperatively contribute to the BRAFi resistance in BRAF-mutated melanoma cells, implying the potential of laminB1 in promoting efficacy of targeted therapies.

## Data Availability

The data that support the findings of this study are available upon reasonable request.

## Supplementary Materials

The following supporting information can be downloaded at: https://www.mdpi.com/article/10.3390/cancers16234060/s1, File S1: Original western blots.
