# Supplementary material for: Interfering Nuclear Protein Laminb1 Induces DNA Damage and Reduces Vemurafenib Resistance in Melanoma Cells In Vitro
Source: Cancers (Basel). 2024 Dec 4;16(23):4060. doi: 10.3390/cancers16234060 (PMC11639818; doi:10.3390/cancers16234060)
Supplement: Supplementary file 1 [file cancers-16-04060-s001.zip › cancers-3317071-supplementary.pdf]

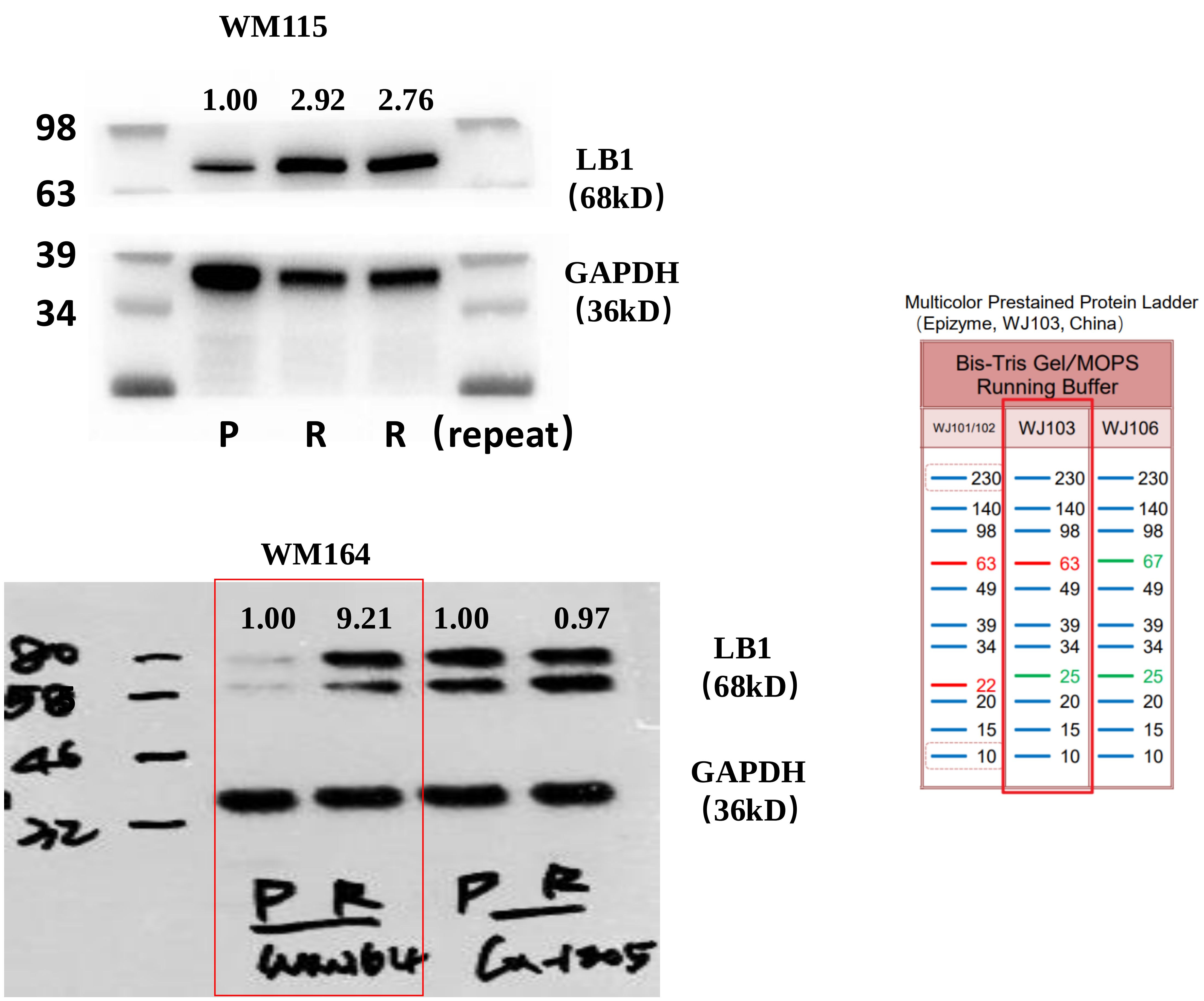

Fig S1: The original and uncropped version of the western blot in Figure 2B.

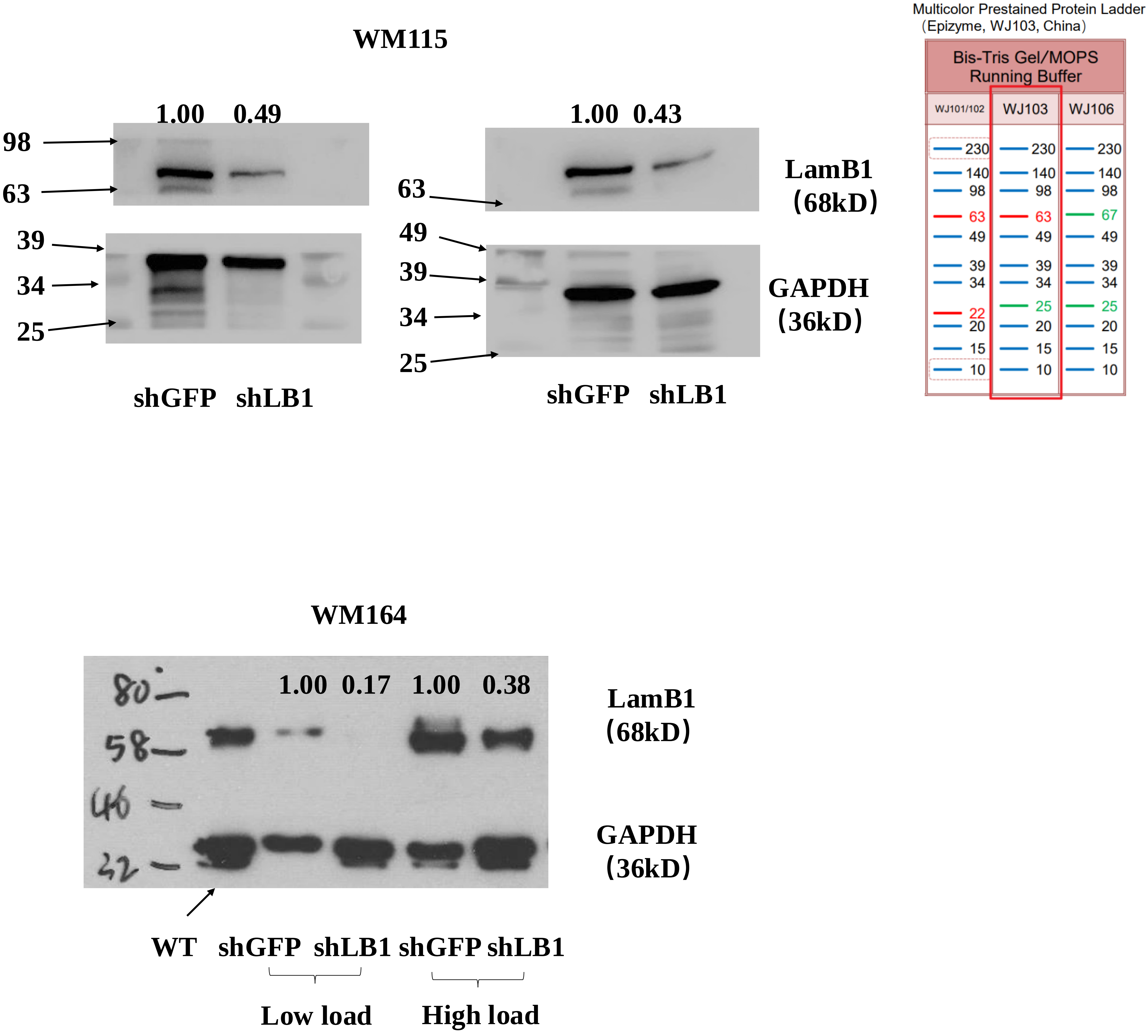

Fig S2: The original and uncropped version of the western blot in Figure 2D.

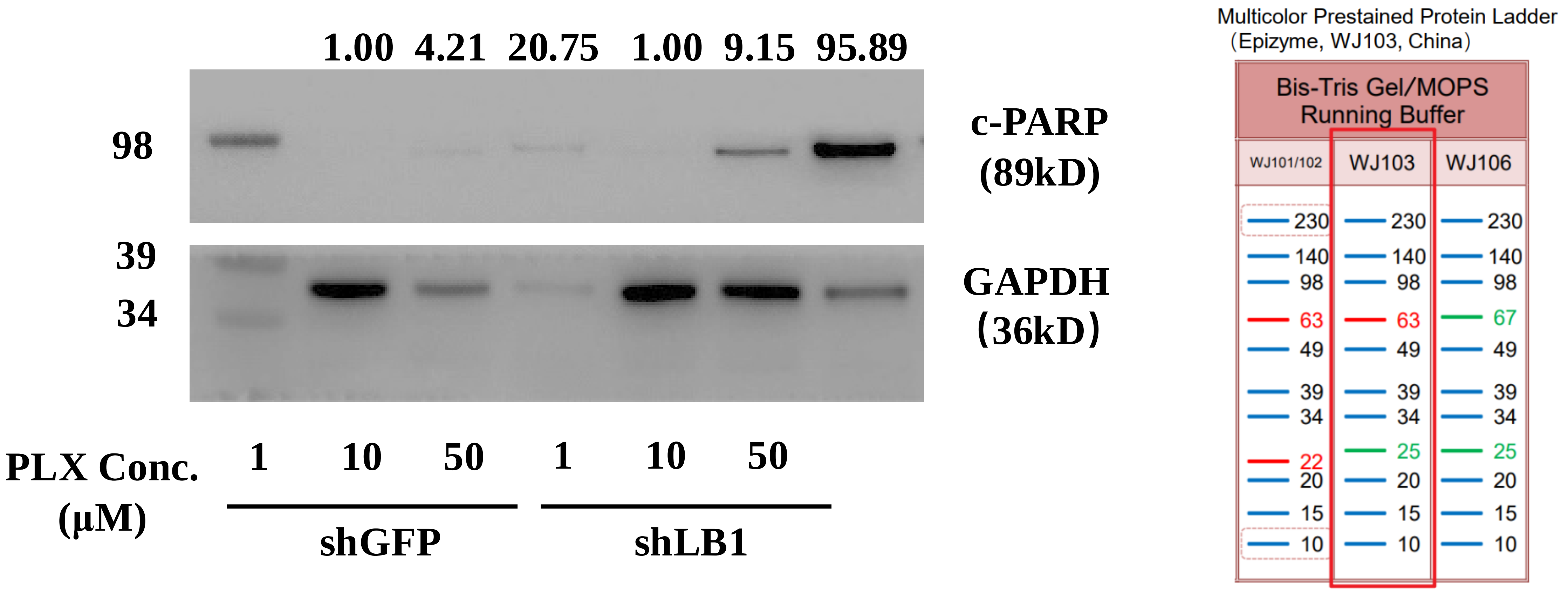

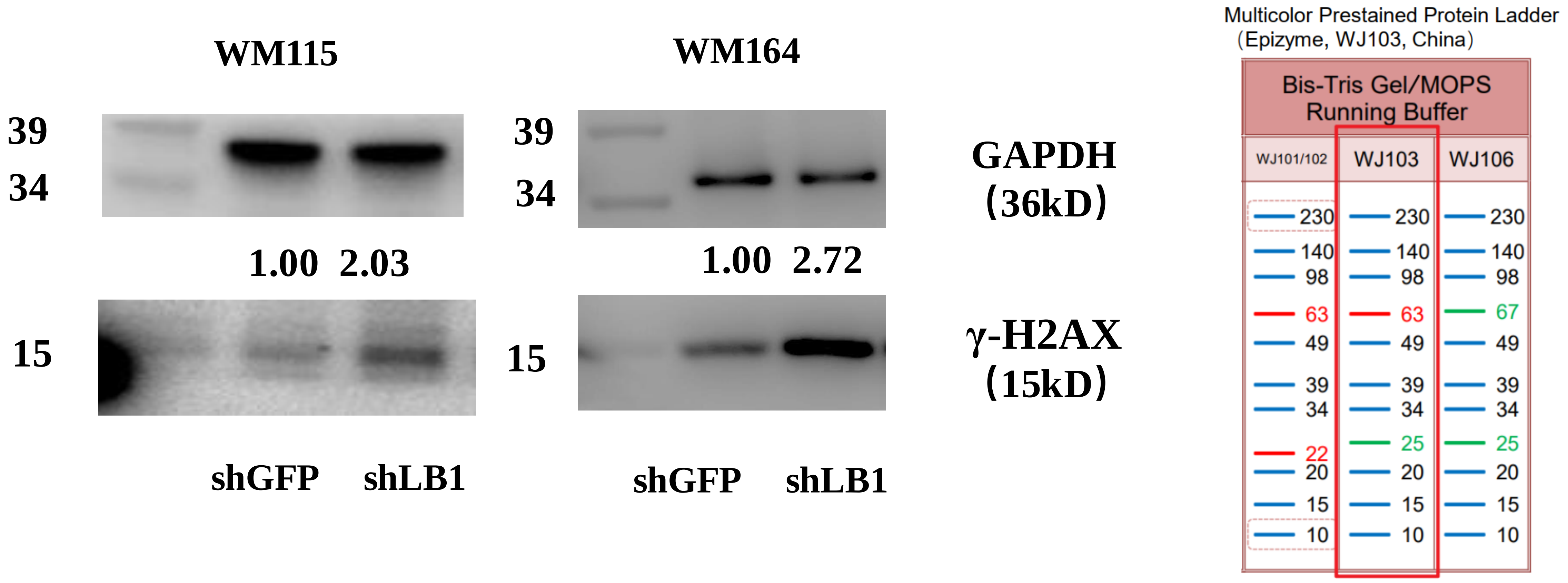

Fig S3: The original and uncropped version of the western blot in Figure 3A (Top) and Figure 3C (Bottom).

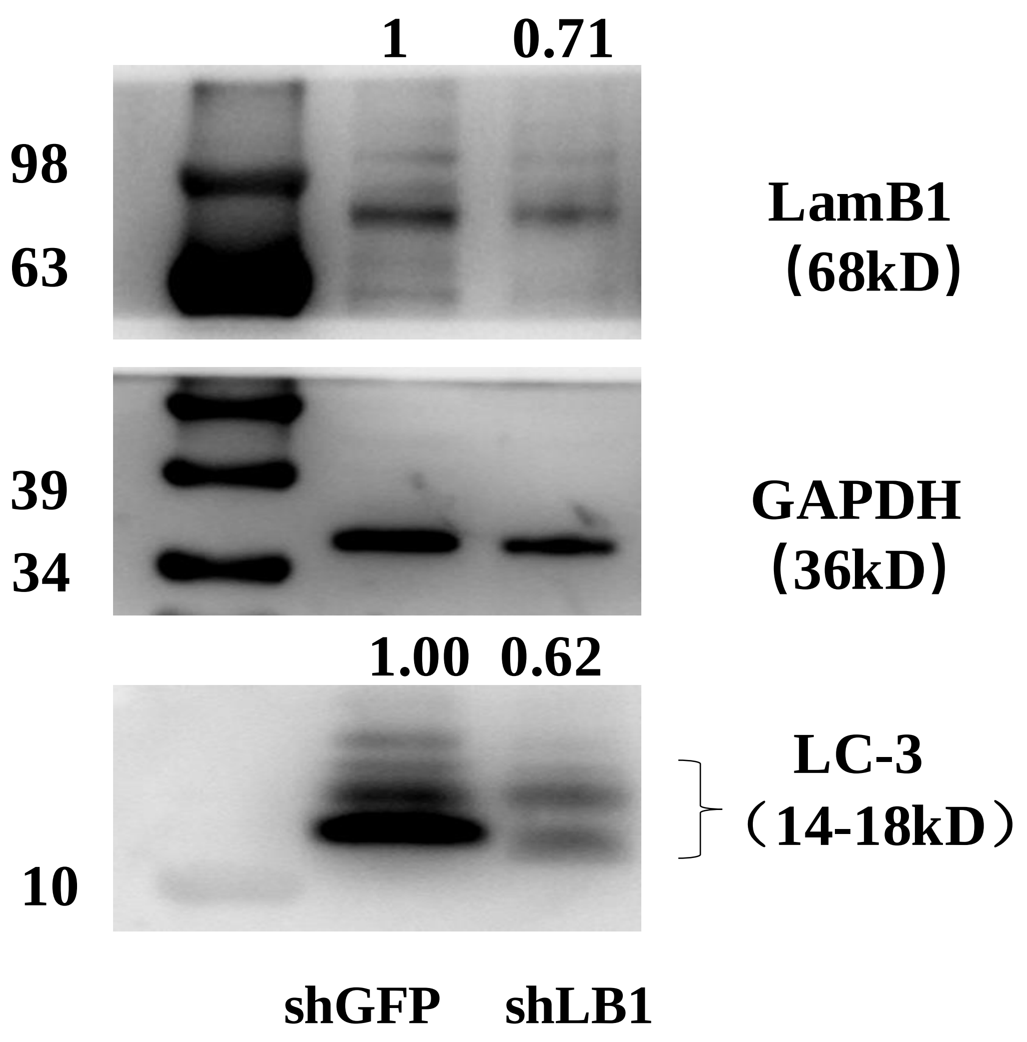

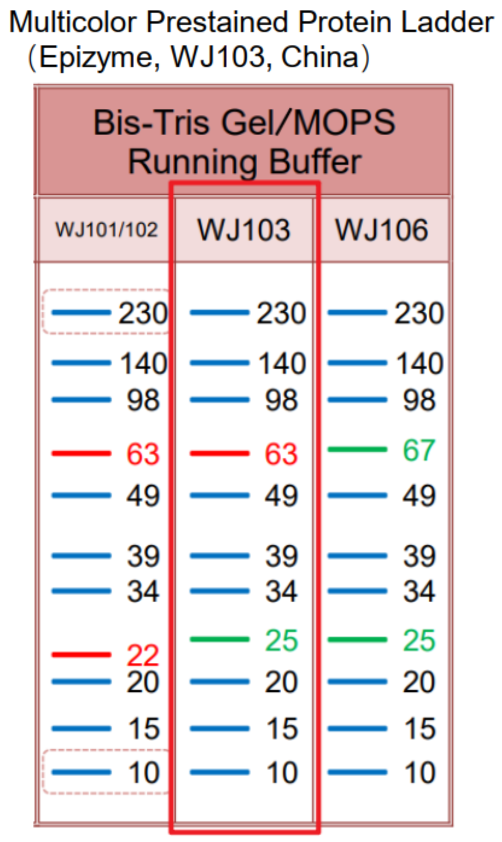

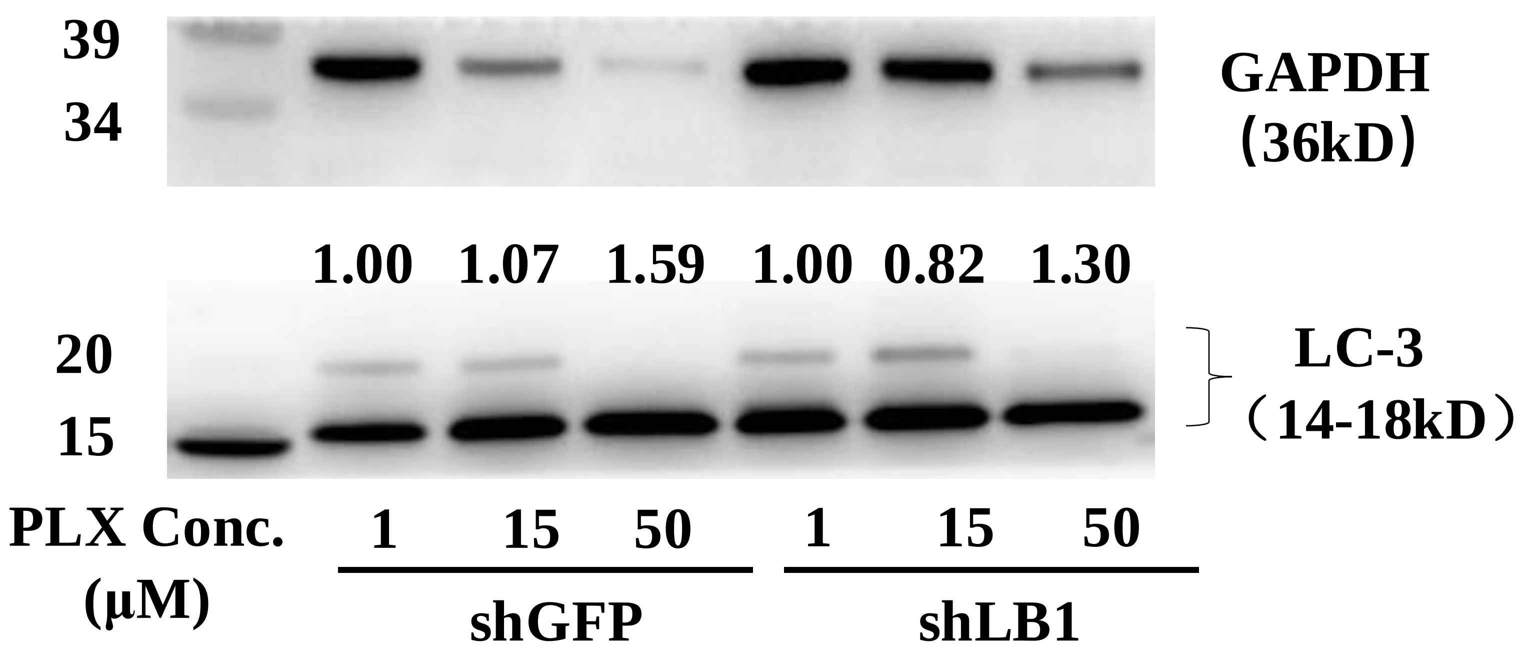

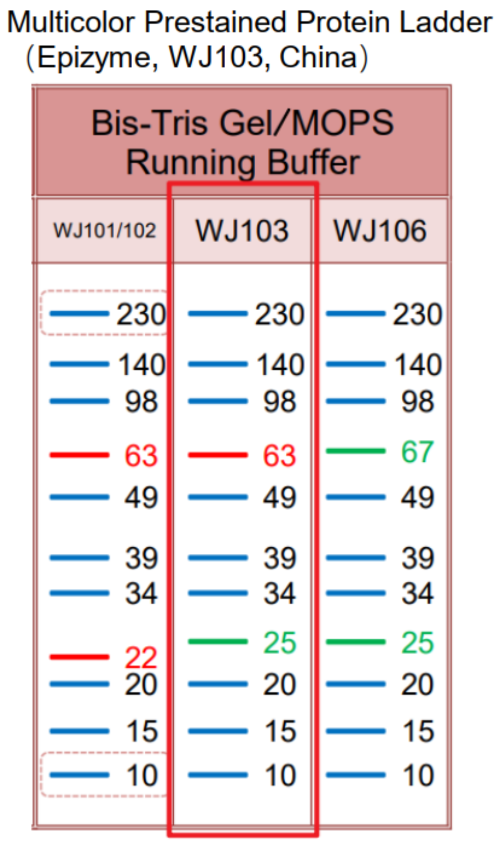

Fig S4: The original and uncropped version of the western blot in Figure 5A (Top) and Figure 5B (Bottom).
